# Conscious sedation/monitored anesthesia care versus general anesthesia in patients undergoing transcatheter aortic valve replacement: A meta-analysis

**DOI:** 10.3389/fcvm.2022.1099959

**Published:** 2023-01-10

**Authors:** Kuo-Chuan Hung, Jen-Yin Chen, Chung-Hsi Hsing, Chin-Chen Chu, Yao-Tsung Lin, Yu-Li Pang, I-Chia Teng, I-Wen Chen, Cheuk-Kwan Sun

**Affiliations:** ^1^School of Medicine, College of Medicine, National Sun Yat-sen University, Kaohsiung City, Taiwan; ^2^Department of Anesthesiology, Chi Mei Medical Center, Tainan City, Taiwan; ^3^Department of Medical Research, Chi Mei Medical Center, Tainan City, Taiwan; ^4^Department of Anesthesiology, Chi Mei Medical Center, Liouying, Tainan City, Taiwan; ^5^Department of Emergency Medicine, E-Da Hospital, Kaohsiung City, Taiwan; ^6^College of Medicine, I-Shou University, Kaohsiung City, Taiwan

**Keywords:** aortic stenosis, conscious sedation, monitored anesthesia care, prognostic outcomes, transcatheter aortic valve replacement, meta-analysis

## Abstract

**Background:**

To compare the merits and safety between conscious sedation/monitored anesthesia (CS/MAC) and general anesthesia (GA) for patients receiving transcatheter aortic valve replacement (TAVR).

**Measurements:**

Databases including EMBASE, MEDLINE, and the Cochrane Library databases were searched from inception to October 2022 to identify studies investigating the impact of CS/MAC on peri-procedural and prognostic outcomes compared to those with GA. The primary outcome was the association of CS/MAC with the risk of 30-day mortality, while secondary outcomes included the risks of adverse peri-procedural (e.g., vasopressor/inotropic support) and post-procedural (e.g., stroke) outcomes. Subgroup analysis was performed based on study design [i.e., cohort vs. matched cohort/randomized controlled trials (RCTs)].

**Main results:**

Twenty-four studies (observational studies, *n* = 22; RCTs, *n* = 2) involving 141,965 patients were analyzed. Pooled results revealed lower risks of 30-day mortality [odd ratios (OR) = 0.66, *p* < 0.00001, 139,731 patients, certainty of evidence (COE): low], one-year mortality (OR = 0.72, *p* = 0.001, 4,827 patients, COE: very low), major bleeding (OR = 0.61, *p* = 0.01, 6,888 patients, COE: very low), acute kidney injury (OR = 0.71, *p* = 0.01, 7,155 patients, COE: very low), vasopressor/inotropic support (OR = 0.25, *p* < 0.00001, 133,438 patients, COE: very low), shorter procedure time (MD = −12.27 minutes, *p* = 0.0006, 17,694 patients, COE: very low), intensive care unit stay (mean difference(MD) = −7.53 h *p* = 0.04, 7,589 patients, COE: very low), and hospital stay [MD = −0.84 days, *p* < 0.00001, 19,019 patients, COE: very low) in patients receiving CS/MAC compared to those undergoing GA without significant differences in procedure success rate, risks of cardiac-vascular complications (e.g., myocardial infarction) and stroke. The pooled conversion rate was 3.1%. Results from matched cohort/RCTs suggested an association of CS/MAC use with a shorter procedural time and hospital stay, and a lower risk of vasopressor/inotropic support.

**Conclusion:**

Compared with GA, our results demonstrated that the use of CS/MAC may be feasible and safe in patients receiving TAVR. However, more evidence is needed to support our findings because of our inclusion of mostly retrospective studies.

**Systematic review registration:**

https://www.crd.york.ac.uk/prospero/, identifier CRD42022367417.

## 1. Introduction

The incidence of aortic valve stenosis, which is one of the most common acquired valvular heart disorders in the aged population, has risen with an increased life expectancy (1). In addition to early recognition, timely treatment is critical for the improvement of survival rate (2). Compared with conventional surgical aortic valve replacement, transcatheter aortic valve replacement (TAVR) not only has a lower early (3) and all-cause mortality as well as incidence of stroke up to 2 years (4) but it is also indicated for high-risk patients with severe symptomatic aortic stenosis deemed unsuitable for conventional surgery (3–6).

To enable the operation of transesophageal echocardiography and prompt surgical interventions for various complications, TAVR is typically conducted under general anesthesia (GA) which, however, is associated with the risks of intraoperative hemodynamic instability that required the use of inotropic agents (7) as well as potential postoperative respiratory complications (8). To minimize such adverse impacts on patient’s outcomes, previous observational studies have shown the feasibility of using local anesthesia, conscious sedation (CS), and monitored anesthesia care (MAC) for the procedure (9–13). Nevertheless, the impact of different anesthetic approaches on prognostic outcomes (e.g., 30-day mortality rate) in patients undergoing TAVR remains inconsistent. While two meta-analyses of observational studies (14, 15) reported a lower 30-day mortality with the use of local anesthesia as the main anesthetic strategy compared to that with GA, a recent randomized controlled trial (RCT) demonstrated no difference in mortality between patients receiving GA and those undergoing local anesthesia/CS (16).

A number of previous observational studies have reported comparable 30-day mortality between patients who underwent TAVR under GA and those receiving CS/MAC (10, 17), despite the demonstration of a shorter stay in the intensive care unit (ICU) (13, 18, 19) and hospital length of stay (LOS) (10, 17). On the other hand, one large-scale retrospective study of 10,997 patients undergoing TAVR under sedation or GA showed that those receiving “conscious sedation” may have a reduced 30-day mortality (18). Therefore, compared with GA, TAVR under CS/MAC appeared to be associated with a shorter ICU stay or LOS with similar or potentially reduced 30-day mortality. Nevertheless, there is a lack of evidence supporting the merits and safety of CS/MAC as the main anesthetic techniques from a systematic approach in the current literature. Taking into account the inconsistent positive impacts of CS/MAC on post-TAVR outcomes (i.e., 30-day mortality rate) (8, 20, 21) and the increasing use of CS/MAC in recent years (22, 23), a meta-analytical investigation into current literature may provide evidence that helps optimizing patient care for clinicians. We hypothesized that the use of CS/MAC would offer patient outcomes comparable to those with GA. The primary outcome of the current meta-analysis was the risk of 30-day mortality, while the secondary outcomes included peri-procedural outcomes (e.g., procedure success rate and procedural time), risks of cardiovascular complications and stroke, as well as medical resource utilization [e.g., hospital LOS].

## 2. Materials and methods

The protocol for the current meta-analysis, which we posted online, was registered in PROSPERO (CRD42022367417). All procedures of the current study complied with the reporting recommendations from Preferred Reporting Items for Systematic Reviews and Meta-Analyses (PRISMA).

### 2.1. Data sources and searches

Using a combination of keywords and Medical Subject Headings (MeSH) terms, we searched the electronic databases of EMBASE, Medline, and the Cochrane Central Register of Controlled Trials from inception to October 7, 2022. The details of our search strategies are available in Supplementary Table 1. The keywords included (“transcatheter aortic valve replacement” or “TAVR” or “Aortic valve stenosis” or “Transcatheter Aortic Valve Implantation (TAVI)” or “TAVI”) and (“General anesthesia” or “Tracheal intubation*” or “Endotracheal Intubation”) and (“Sedation” or “Monitored anesthesia care”). Neither the language nor the publication year were subjected to any restrictions, but studies with a sample size < 200 before propensity matched analysis were excluded to reduce the possibility of underpowering as previously reported (24). Additionally, we performed a manual search of the references of relevant review articles and each study included in our analysis to identify other records that we may have missed during our initial search.

### 2.2. Inclusion and exclusion criteria

We included randomized and observational studies that met the PICO (i.e., population, intervention, comparator, and outcome) criteria: (1) Population: adults receiving TAVR regardless of the products and techniques; (2) Intervention: the use of CS or MAC as the main anesthetic approach with or without the involvement of anesthesiologists/anesthesia nurses, combined use of local anesthetics, and nerve block; (3) Comparator: the use of GA regardless of anesthetic agents (e.g., inhalation agents and propofol) or airway techniques (e.g., tracheal intubation or insertion of laryngeal mask airway); and (4) Outcome: available prognostic outcomes such as risk of mortality or hospital LOS.

The exclusion criteria were (1) studies with a sample size < 200 before propensity matched analysis; (2) those focused on local anesthesia as the main anesthetic technique; (3) those in which detailed information on anesthetic techniques or outcomes of comparison between CS/MAC and GA was unavailable; (4) studies published only as letters, abstracts, or review articles; (5) those included participants younger than 18 years.

### 2.3. Studies selection and data extraction

After removing duplicate records, two reviewers independently determined the inclusion or exclusion of the articles based on the titles and abstracts according to the PICO criteria. The full text of the articles was further reviewed to evaluate their eligibility. Discussion with a third party resolved discrepancies between the independent reviewers. The same procedure was applied to both data extraction and bias assessment. The following details were extracted from the included studies using a standardized checklist: authors/year of publication, population characteristics (e.g., body mass index), study setting (i.e., cohort, matched cohort, and RCTs), number of participants, mortality rate, peri- or post-procedure complications (e.g., bleeding or pacemaker implantation), other prognostic outcomes (e.g., hospital stay or stroke), and country.

### 2.4. Study outcomes and definition

The primary outcome was the risk of 30-day all-cause mortality. For studies that only reported the risk of in-hospital mortality, we used this outcome as an alternative. The secondary end points included the risks of mortality at one year, pacemaker implantation, stroke, acute kidney injury (AKI), myocardial infarction (MI), major bleeding, vascular complications, vasopressor/inotropic support, and procedural time, ICU/hospital LOS as well as procedure success rate defined as successful device deployment.

### 2.5. Quality assessment of included studies and certainty of evidence

Two reviewers independently assessed the risk of bias of RCT by using standard criteria defined in the Cochrane Handbook for Systematic Reviews of Interventions (i.e., ROB 2.0) and the Newcastle-Ottawa Scale (NOS) for non-randomized studies as previously reported (25). The overall certainty of evidence for primary and secondary outcomes was assessed based on the Grading of Recommendations Assessment, Development and Evaluation (GRADE) framework. Discrepancies regarding overall certainty of evidence were settled through discussion.

### 2.6. Data synthesis and analysis

Based on a random-effects model, pooled data are presented as odds ratio (OR) and mean difference (MD) with 95% confidence interval (CI) for dichotomous and continuous data, respectively. Subgroup analysis was performed according to the study design (i.e., cohort vs. matched cohort/RCT). We combined matched observational studies and RCTs for analysis based on their similar characteristics of patient matching for preventing bias. We assessed the heterogeneity of pooled effects with Higgins I^2^ with substantial heterogeneity being defined as an I^2^ over 50% as previously reported (26, 27). The reliability and conclusiveness of evidence generated was examined with sensitivity analysis through omitting one study each time. On encountering 10 or more trials that shared a particular outcome, we evaluated the potential publication bias by visual inspection of a funnel plot. A p value of < 0.05 was deemed statistically significant in the current meta-analysis. All statistical analyses were performed using the comprehensive Meta-Analysis (CMA) V3 software (Biostat, Englewood, NJ, USA) or Review Manager (RevMan) computer program, version 5.3.5 (The Nordic Cochrane Centre, The Cochrane Collaboration, Copenhagen, 2014).

## 3. Results

### 3.1. Study selection and characteristics

**FIGURE 1:**
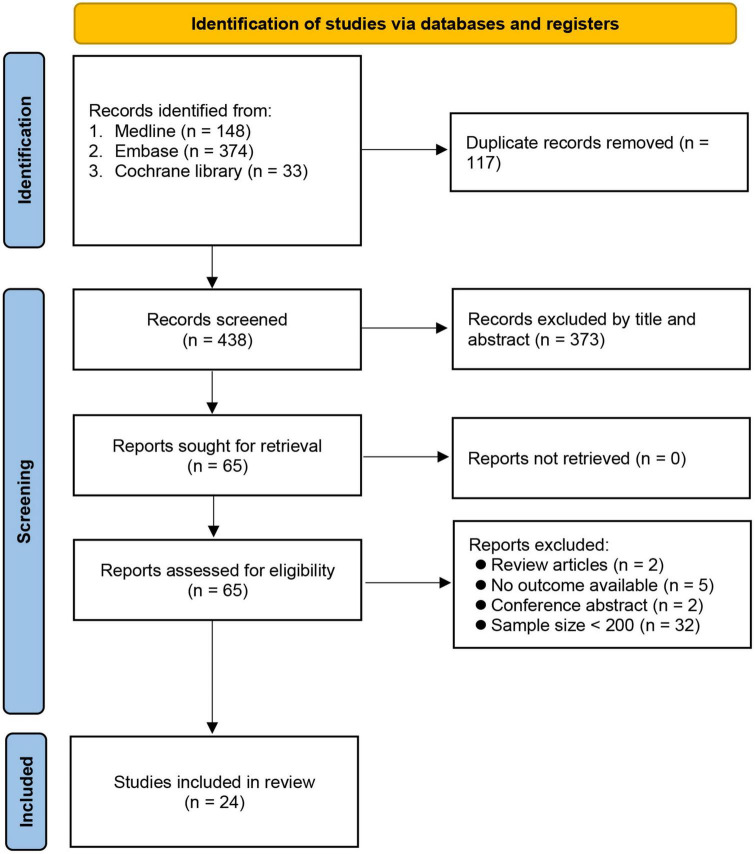
PRISMA flow diagram of study selection for the current meta-analysis.

The initial search identified 555 potential articles. After removing duplicates (*n* = 117) and records not meeting our inclusion criteria after title and abstract screening (*n* = 373), we conducted a full-text review on 65 articles. Forty studies were further excluded because of being review articles (*n* = 2) and conference abstracts (*n* = 2), unavailability of data on outcome (*n* = 5), and a sample size < 200 (*n* = 32). Finally, 24 studies involving 141,965 patients published between 2015 and 2021 were included for analysis (7, 8, 16, 18–23, 28–42). The process of study selection is shown in Figure 1.

The mean or median age of the enrolled patients receiving TAVR varied from 73 to 83 years with the proportion of males ranging from 36% to 99%. One study did not provide information regarding male proportion (35). The design of the eligible articles included retrospective studies (cohort, *n* = 12; matched cohort, *n* = 9) (7, 8, 18–23, 28–30, 32–40, 42), prospective observational study (*n* = 1) (41), and RCT (*n* = 2) (16, 31). Although two articles were considered separate RCTs (16, 31), they shared the same patient population but focused on different time points of outcome assessment [i.e., 30-day (16) and one-year (31)]. Another retrospective study utilized data from two previous observational studies that compared TAVR with conventional surgical valve replacement in patients with an intermediate (43) and a low (44) surgical risk. One study initially included 304 patients for assessment, but the number dropped to 162 after propensity matched analysis (36). The size of the study population varied widely from 162 to 120,080 in the included studies. The mean or median Society of Thoracic Surgeons (STS) predicted risk for mortality score ranged from 1.8 to 11 in seventeen studies (16, 19, 20, 22, 29, 31–42), while another seven did not provide relevant detail. The CS/MAC group had a lower STS score compared to that in the GA group (MD = −0.46, 95% CI: −0.77 to −0.16) (Supplementary Figure 1). Fifteen studies involving 70,246 patients provided information on the conversion rate of CS/MAC to GA, which ranged from 0 to 12% with a pooled incidence of 3.1% (95% CI 1.8% to 5.4%) (Supplementary Figure 2). Nevertheless, only six of the 15 studies described their allocation of patients undergoing conversion (i.e., CS/MAC to GA) to the CS/MAC group based on the intention-to-treat principle, while the other nine studies did not provide relevant information. Of our 24 included studies, 12 provided details regarding the assessment of aortic regurgitation in the CS/MAC group by using transthoracic echocardiography, fluoroscopic guidance, or transesophageal echocardiography under sedation (Supplementary Table 2). These included studies were conducted in seven countries, namely USA (*n* = 14), United Kingdom (*n* = 2), Germany (*n* = 4), Italy (*n* = 1), Israel (*n* = 1), Korea (*n* = 1), and France (*n* = 1). The NOS score of the 22 observational studies ranged from 7 to 8 (Table 1 and Supplementary Table 3), suggesting a low risk of bias. The overall risk of bias in the other two RCTs were also considered low (Supplementary Table 4).

**TABLE 1:** Studies characteristics of included studies (*n* = 24).

References	Study year	Age (year)*	Male (%)*	BMI (kg/m^2^)*	*N*	Mean or median STS score*	Study design	Conversion rate	Country	Nos
Abbett et al. (28)	2013–2018	81 vs. 81	60 vs. 60	29 vs. 29	318	NA	MC	NA	USA	8
Ahmad et al. (19)	2012–2018	80 vs. 82	51 vs. 54	20 vs. 16	418	5.7 vs. 8.4	C	NA	USA	7
Brecker et al. (20)	2010–2011	81 vs. 82	49 vs. 47	NA	490	5.3 vs. 5.2	MC	5.3%	United Kingdom	8
Burns et al. (29)	2014–2017	82 vs. 80	55 vs. 68	30 vs. 29	214	3.6 vs. 3.6	MC	0	USA	8
Butala et al. (23)	2016–2019	81-82^†^	54^†^	28.0^†^	120080	NA	C	1.3%	USA	8
D’Errigo et al. (21)	2010–2012	83 vs. 82	65 vs. 62	26 vs. 26	620	NA	MC	NA	Italy	8
Eskandari et al. (30)	2013–2014	82 vs. 81	53 vs. 54	27 vs. 28	306	NA	MC	NA	United Kingdom	8
Feistritzer et al. (31)	2016–2018	82^†^	48.9^†^	NA	438	4.5 vs. 5.1	RCT	NA	Germany	−^§^
Goren et al. (7)	2009–2012	83 vs. 83	40 vs. 36	27 vs. 28	204	NA	C	4.7%	Israel	7
Herrmann et al. (33) ^‡⁣‡^	2014 2016–2017	82 vs. 82 74 vs. 73	63 vs. 60 69 vs. 66	28 vs. 29 30 vs. 31	950 493	5.2 vs. 5.3 2 vs. 1.8	C C	1.7% 2.4%	USA USA	7 7
Hyman et al. (18)	2014–2015	82 vs. 82	54 vs. 54	28 vs. 29	10997	NA	C	5.9%	USA	7
Harjai et al. (32)	2014–2018	83 vs. 82	51 vs. 49	29 vs. 29	477	5 vs. 5	C	3%	USA	7
Kiramijyan et al. (34)	2007–2015	83 vs. 81	51 vs. 50	27 vs. 31	533	8.5 vs. 9.8	C	12%	USA	7
Kislitsina et al. (35)	2012–2016	81 vs. 82	NA	NA	286	6 vs. 8.4	C	3.3%	USA	7
Lau et al. (36)	2013–2017	83 vs. 80	49 vs. 54	29 vs. 30	162^‡^	5.1 vs. 5.7	MC	2.3%	USA	8
Lee et al. (8)	2011–2019	80 vs. 78	49 vs. 52	24.vs 24	589	NA	C	NA	Korea	7
Lum et al. (37)	2013–2019	78 vs. 80	99 vs. 96	NA	227	3.0 vs. 4.9	MC	1.55%	USA	8
Mosleh et al. (38)	2012–2018	82 vs. 80	47 vs. 58	27 vs. 30	308	10.3 vs. 11	MC	1%	USA	8
Musuku et al. (39)	2017–2019	83 vs. 81	48 vs. 55	26 vs. 28	296	4.8 vs. 4.2	MC	NA	USA	8
Neumann et al. (40)	2014–2015	82 vs. 81	50 vs. 52	NA	1694	7.4 vs. 7	C	2.9%	Germany	7
Renner et al. (41)	2012–2014	82 vs. 82	52 vs. 41	26 vs. 26	200	6.6 vs. 6.2	PO	4.3%	Germany	8
Sammour et al. (22)	2012–2017	81 vs. 80	59 vs. 56	29 vs. 29	998	6.7 vs. 7.6	C	NA	USA	7
Thiele et al. (16)	2016–2021	82 vs. 81	49 vs. 49	26 vs. 27	438	4.5 vs. 5.1	RCT	6.0%	Germany	−^§^
Zaouter et al. (42)	2013–2014	82 vs. 80	49 vs. 51	27 vs. 26	229	7.5 vs. 7.8	C	NA	France	7

C, cohort; MC, matched cohort; R, randomized controlled studies; PO, prospective observational; ^†^overall patients; NOS, Newcastle-Ottawa Scale; STS score, society of thoracic surgeons risk score; ^‡^a total of 304 patients included before propensity matched analysis; ^§^assessment by using ROB 2.0; NA, not available; BMI, body mass index; *presented as conscious sedation/monitored anesthesia care group vs. general anesthesia group; ^‡⁣‡^two dataset available.

### 3.2. Synthesis of results

#### 3.2.1. Impact of CS/MAC on risk of 30-day and one-year mortality

**FIGURE 2:**
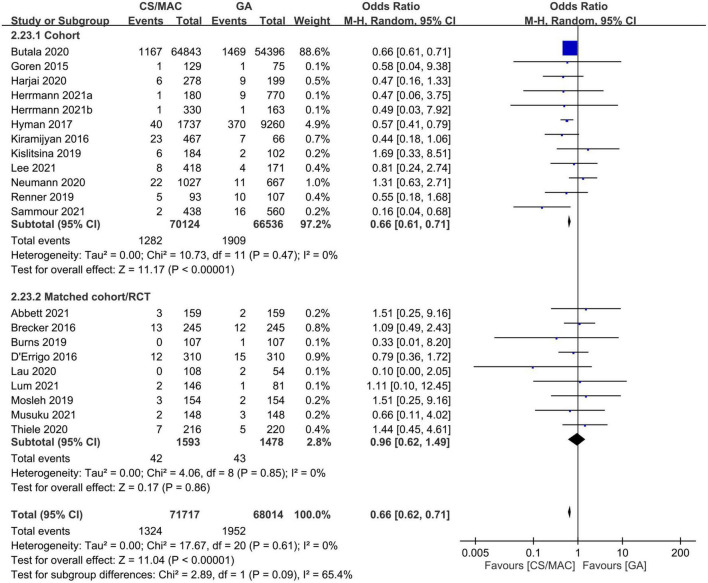
Forest plot comparing the risk of 30-day mortality between patients receiving conscious sedation/monitored anesthesia care (CS/MAC) and those undergoing general anesthesia (GA). CI, confidence interval; M-H, Mantel-Haenszel.

Twenty studies are available for analysis of the impact of CS/MAC on the risk of 30-day mortality (Figure 2). Pooled results revealed a lower risk of 30-day mortality in patients receiving CS/MAC compared to that in those receiving GA (OR = 0.66, 95% CI: 0.62 to 0.71, *p* < 0.00001, I^2^ = 0%, 139,731 patients, 20 studies) with consistent findings on sensitivity analysis. Funnel plot showing a low risk of publication bias (Supplementary Figure 3). Subgroup analysis focusing on study design (i.e., cohort vs. matched cohort/RCT) demonstrated no significant impact of matched cohort/RCT (OR = 0.0.96, 95% CI: 0.62 to 1.49, *p* = 0.86, I^2^ = 0%, 3071 patients, 9 studies) on the 30-day mortality risk between CS/MAC and GA, despite persistent significance for a cohort design (Figure 2).

Regarding one-year mortality risk, our findings also showed a correlation between a lower risk of one-year mortality and the use of CS/MAC compared to GA (OR = 0.72, 95% CI: 0.59 to 0.88, *p* = 0.001, I^2^ = 5%, 4,827 patients, 9 studies). The results remained consistent on sensitivity analysis (Supplementary Figure 4). Again, despite no significant influence of a matched cohort/RCT design on the one-year mortality risk between CS/MAC and GA (OR = 0.86, 95% CI: 0.61 to 1.2, *p* = 0.37, I^2^ = 0%, 1,090 patients, 3 studies), the effect was significant for cohort studies on subgroup analysis. Therefore, the findings did not provide a robust support for the association between CS/MAC and a reduced risk of one-year mortality.

#### 3.2.2. Impact of CS/MAC on risk of cardiac complications

There was no difference in the risk of MI (OR = 1.07, 95% CI: 0.43 to 2.65, *p* = 0.88, I^2^ = 0%, 4,830 patients, 8 studies) (Supplementary Figure 5) and pacemaker implantation (OR = 1.05, 95% CI: 0.9 to 1.22, *p* = 0.56, I^2^ = 30%, 18516 patients, 16 studies) (Supplementary Figure 6) between CS/MAC and GA groups. Sensitivity and subgroup analysis revealed consistent findings on these two outcomes, suggesting robustness of evidence. Funnel plot showed a low risk of publication bias regarding the outcome of pacemaker implantation (Supplementary Figure 7).

#### 3.2.3. Impact of CS/MAC on procedure-related outcomes and vasopressor/Inotropic support

Pooled results demonstrated a shorter procedural time in the CS/MAC group than that in the GA group (MD = −12.27 min, 95% CI: −19.24 to −5.31, *p* = 0.0006, I^2^ = 95%, 17,694 patients, 15 studies) with sensitivity analysis showing consistent finding (Supplementary Figure 8). Subgroup analysis revealed a consistent result on matched cohort/RCT studies, but not in cohort studies that showed no difference in procedural time. Nevertheless, the funnel plot indicated a potential risk of publication bias (Supplementary Figure 9).

There was no difference in risk of vascular complications between the two groups (OR = 1.1, 95% CI: 0.89 to 1.37, *p* = 0.38, I^2^ = 0%, 6,930 patients, 13 studies) with unaverred results on sensitivity and subgroup analysis (Supplementary Figure 10). However, the funnel plot suggested a potential risk of publication bias (Supplementary Figure 11).

On the other hand, the use of CS/MAC was associated with a lower risk of major bleeding compared to that with GA (OR = 0.61, 95% CI: 0.41 to 0.9, *p* = 0.01, I^2^ = 58%, 6,888 patients, 13 studies). The finding remained consistent on sensitivity analysis (Supplementary Figure 12). Subgroup analysis also indicated a lower risk of major bleeding related to the use of CS/MAC in cohort studies, but no difference in risk was noted in matched cohort/RCT studies. Funnel plot suggested a low risk of publication bias (Supplementary Figure 13).

There was no difference in procedure success rate between the CS/MAC group (97.5%) and the GA group (97.4%) (OR = 1.02, 95% CI: 0.71 to 1.47, *p* = 0.92, I^2^ = 61%, 132,391 patients, 7 studies) with consistency of the finding being supported by sensitivity and subgroup analyses (Supplementary Figure 14).

**FIGURE 3:**
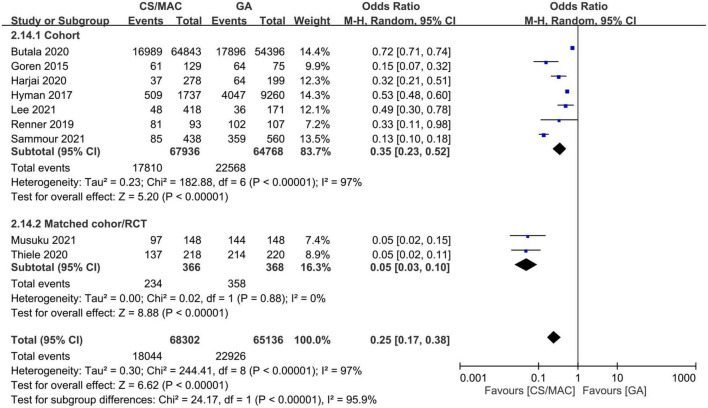
Forest plot comparing the risk of vasopressor/inotropic support in patients receiving conscious sedation/monitored anesthesia care (CS/MAC) or general anesthesia (GA). CI, confidence interval; M-H, Mantel-Haenszel.

The use of CS/MAC correlated with a lower risk of vasopressor/inotropic support compared to that with GA (OR = 0.25, 95% CI: 0.17 to 0.38, *p* < 0.00001, I^2^ = 97%, 133,438 patients, 9 studies). The result was consistent on sensitivity and subgroup analyses (Figure 3).

Taken together, our results demonstrated robust evidence supporting a lack of difference in the procedure success rate and the risk of vascular complications between the CS/MAC and GA groups. On the other hand, despite a shorter procedural time and a lower risk of major bleeding associated with CS/MAC compared to GA, our subgroup analysis did not endorse the soundness of these findings.

#### 3.2.4. Impact of sedation on risk of stroke and acute kidney injury

**FIGURE 4:**
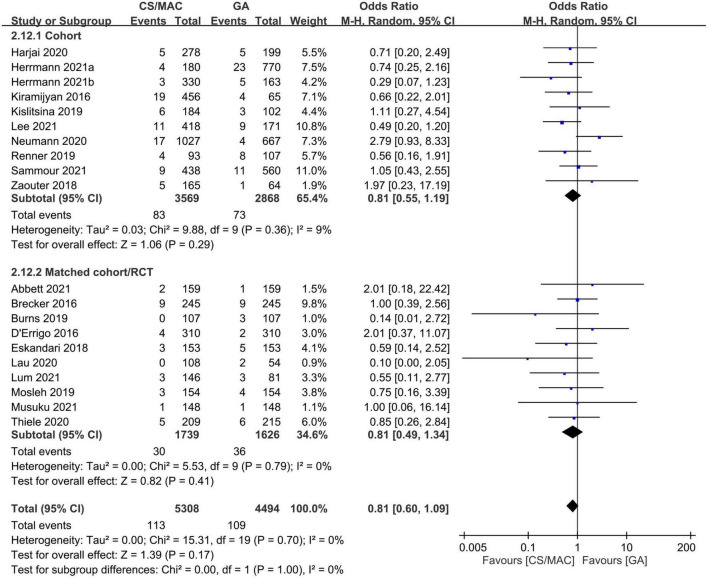
Forest plot comparing the risk of stroke in patients receiving conscious sedation/monitored anesthesia care (CS/MAC) or general anesthesia (GA). CI, confidence interval; M-H, Mantel-Haenszel.

Our pooled results that showed no significant difference in the risk of stroke between the CS/MAC and GA groups (OR = 0.81, 95% CI: 0.6 to 1.09, *p* = 0.17, I^2^ = 0%, 9,802 patients, 20 studies) remained consistent on sensitivity and subgroup analyses (Figure 4). In addition, the funnel plot indicated a low risk of publication bias (Supplementary Figure 15).

The CS/MAC group demonstrated a lower risk of AKI compared to that in the GA group (OR = 0.71, 95% CI: 0.54 to 0.92, *p* = 0.01, I^2^ = 0%, 7,155 patients, 15 studies). However, the result was inconsistent on sensitivity and subgroup analyses (Supplementary Figure 16). An inspection of the funnel plot suggested a low risk of publication bias (Supplementary Figure 17).

Overall, the current study indicated robust evidence that suggested no significant difference between CS/MAC and GA in their association with the risk of stroke, while there was weak evidence that implied a potential correlation between the use of CS/MAC and a lower risk of AKI compared with GA.

#### 3.2.5. Impact of sedation on medical resource utilization

The use of CS/MAC was associated with a shorter ICU stay compared to GA (MD = −7.53 h, 95% CI: −14.82 to −0.25, *p* = 0.04, I^2^ = 100%, 7,589 patients, 14 studies) (Supplementary Figure 18) with inconsistent findings on sensitivity and subgroup analyses. Funnel plot demonstrated a low risk of publication bias (Supplementary Figure 19).

**FIGURE 5:**
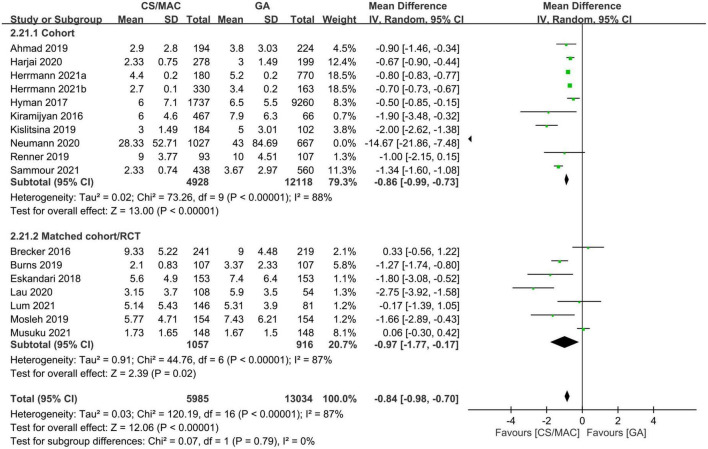
Forest plot comparing the hospital length of stay between patients receiving conscious sedation/monitored anesthesia care (CS/MAC) and those subjected to general anesthesia (GA). CI, confidence interval; M-H, Mantel-Haenszel.

There was a shorter hospital LOS in the sedation group compared to that in the GA group (MD = −0.84 days, 95% CI: −0.98 to −0.7, *p* < 0.00001, I^2^ = 87%, 19,019 patients, 17 studies). The finding remained consistent on sensitivity and subgroup analyses (Figure 5). Besides, examination of the funnel plot indicated a low risk of publication bias (Supplementary Figure 20).

In other words, our analysis suggested robust evidence in support of an association between the use of CS/MAC and a shorter hospital LOS; however, evidence regarding the positive impact of CS/MAC on the length of ICU stay was inconsistent.

### 3.3. Certainty of evidence

The overall certainty of evidence for individual outcome is shown in Supplementary Table 5. The overall certainty of evidence was graded as low in two outcomes (i.e., risk of 30-day mortality and pacemaker implantation), while other outcomes were deemed very low. The main reasons for downgrading the evidence were: (a) inclusion of observational data; (b) a wide 95% CI; and (c) a high I^2^ (i.e., over 50%).

## 4. Discussion

Our results suggested lower risks of 30-day, one-year mortality, AKI, major bleeding, vasopressor/inotropic support as well as a shorter procedural time and ICU/hospital stay with the use of CS/MAC compared to those with GA. The pooled conversion rate from CS/MAC to GA was 3.1%. No difference in the risks of stroke, cardiac [i.e., MI and pacemaker implantation) and vascular complications as well as procedure success rate was noted. Nevertheless, because patients in the CS/MAC group had a lower STS score than that in the GA group as well as inconsistent results on subgroup analysis (e.g., risk of 30-day mortality), these findings should be interpreted with caution.

There have been accumulating data from observational studies regarding the benefits and safety among different anesthetic approaches (i.e., GA vs. CS/MAC vs. local anesthesia) for TAVR. Two previous meta-analyses investigating the application of local anesthesia as the main anesthetic strategy reported an association of using local anesthesia with a lower mortality rate compared to that with GA (14, 15), but one recent RCT showed no significant difference in 30-day mortality between patients undergoing TAVR with local anesthesia/CS and those receiving GA (16). Our results suggested that the use of CS/MAC may be associated with a lower risk of 30-day and one-year mortality compared with GA. Although the pooled evidence was deemed weak, our findings at least suggested that CS/MAC was not inferior to GA when focusing on the safety issue after taking into account the comparable risks of cardiac complications (i.e., MI and pacemaker implantation), vascular complications, and stroke between patients receiving CS/MAC and those undergoing GA for TAVR. Despite such a survival advantage, one potential downside associated with CS/MAC may be the inconvenience of conducting transesophageal echocardiography to detect paravalvular leak. Nevertheless, the use of other diagnostic approaches may compensate for the Achilles heel (Supplementary Table 2).

Although the possible explanation for a lower risk of 30-day and one-year mortality in the CS/MAC group compared to that in the GA group in the current study remains obscure because of their similar risks of cardio- and cerebral-vascular complications, one previous meta-analysis has identified the occurrence of AKI after TAVR as a significant risk factor for both 30-day and long-term (i.e., ≥ 3 years) all-cause mortality (45). Together with their findings that hypertension, diabetes mellitus, peripheral artery disease, ventricular ejection fraction < 40%, and the occurrence of major bleeding were predictors of post-TAVR AKI (45), our results that showed an association of CS/MAC with lower risks of both AKI and major bleeding in patients undergoing CS/MAC may help explain their lower risk of mortality compared to those receiving GA. On the other hand, our finding of a lack of significant influence of the matched cohort/RCT design on subgroup analyses of the 30-day and one-year mortality risks between CS/MAC and GA may be attributed to the relatively small sample sizes (i.e., 3071 and 1090 participants for 30-day and one-year mortality, respectively).

Contrary to the belief that GA is superior to non-GA approaches for TAVR because GA not only can allow patient immobilization to enhance procedural success but also enable the application of transesophageal echocardiography for paravalvular regurgitation assessment as well as facilitate timely management of potential complications (46), several single-institute studies reported comparable TAVR procedural success rate between CS/MAC and GA (41) despite a lack of support from pooled evidence. Consistently, the finding of the current meta-analysis demonstrated no difference in the procedural success rate regardless of the anesthesia approach (i.e., CS/MAC vs. GA), supporting that TAVRs can be performed under CS/MAC without a negative impact on the success rate. Furthermore, an apparently paradoxical finding in the current meta-analysis was the significant but weak evidence showing a shorter procedural time associated with the use of CS/MAC compared to GA. Similarly, two previous meta-analyses have also demonstrated a shorter procedural time with local anesthesia compared to GA (14, 15). One possible explanation may be a coincidence between the learning curve of the TAVR procedure and the choice of anesthesia strategy. While GA was the mainstream of anesthesia at the early stage of TAVR development when the cardiology team was uncertain about the procedure itself and the associated complications, CS/MAC became the anesthesia approach of choice as the team was more adept at the procedure at a later stage (8). Therefore, it is possible that the learning curve of the TAVR procedure, rather than the choice of anesthesia approach, was the cause of the observed reduction in procedural time associated with the use of CS/MAC. The demonstration of no significant difference in procedural time and fluoroscopy time in a recent multicenter RCT comparing the impact of CS and GA on more than 400 patients undergoing TAVRs (16) may reflect the outcome of TAVR as a mature procedure and further support the hypothesis.

Results of prior studies regarding the influence of anesthetic approaches for TAVR on medical resource utilization remain inconsistent. Although a previous meta-analysis on 10,572 patients from 26 studies reported that the use of local anesthesia was associated with a shorter hospital LOS (i.e., 2.09 days) and ICU LOS (i.e., 0.18 days) (14), another recent meta-analysis of 17 studies involving 20,938 patients found no significant difference in the length of total hospital stay between patients receiving local anesthesia and those undergoing GA (15). Our results revealed a shorter ICU LOS (weak evidence) and hospital LOS (strong evidence) with the use of CS/MAC compared to GA. Nevertheless, the reduction in hospital LOS in the current meta-analysis was only 0.84 days with the use of CS/MAC, suggesting no clinically significant improvement from a cost-effective perspective.

There are several merits of the present meta-analysis that may add to the existing knowledge in this field. First, we performed subgroup analysis based on study design (i.e., cohort vs. matched cohort/RCT) to reduce the potential bias arising from variations in patient characteristics on prognostic outcomes. Second, because operator experience and annual TAVR case volume may influence prognostic outcomes (47, 48), we only included studies with a sample size more than 200. This sample size was determined based on the finding of a previous study showing that TAVR performed at a high-volume (i.e., > 100 procedures per year) institute is associated with decreased rates of complications and mortality compared to those with a low annual volume (i.e., < 50 procedures) (47). Therefore, taking into account the observation that most studies collected patient data over a two-year period during our study screening, we adopted a cut-off sample size of over 200 as a threshold for defining a high-volume institute to minimize possible confounding effects from including data of low-volume institutes. Nevertheless, the routine use of CS/MAC for TAVR requires further justifications taking into consideration the impact of technical dexterity on clinical outcomes.

Despite the demonstration of no significant association between vasopressor requirement and anesthetic method (i.e., CS vs. GA) in a small-scale RCT recruiting 62 patients undergoing TAVR (49), most previous studies reported higher requirements of inotropes and/or vasopressors in patients receiving TAVI under GA than those in the MAC group due to GA-related hemodynamic instability (16–18). Consistently, we also found a larger amount of inotrope/vasopressor administered in the GA than in the CS/MAC group. Further studies are required to investigate the impact of periprocedural inotrope/vasopressor use on clinical outcomes.

There were several limitations in the present study. First, meta-analysis of observational data cannot definitively establish the causality of our findings, which requires further RCTs to confirm. Nevertheless, the present study is the first to support the merits and safety of CS/MAC as the main anesthetic technique for patients undergoing TAVI based on clinical evidence derived from a systematic approach. Second, because our most included studies (i.e., 22 retrospective studies) did not specify their criteria for patient grouping, a selection bias (e.g., GA for high-risk patients) cannot be ruled out. Besides, clinicians may tend to adopt GA in the early phase of establishing the TAVR technique and use CS/MAC only in the late phase of procedure development, which may be associated with a maturation of technical skills that contributed to a survival advantage. On the other hand, because our included studies did not specify any chronological sequence of patient allocation in the two groups, such a potential impact cannot be verified. Third, although a previous study reported a reduced cost by 28% with the application of CS/MAC for TAVI (50), this outcome was not analyzed in current meta-analysis because of the limited number of studies available to address this issue. Fourth, most of the included studies were conducted in the United States (Table 1); therefore, extrapolation of our findings to other countries may not be justified. Fifth, previous studies have shown a significant impact of gender on mortality from different approaches to aortic valve replacement. While TAVR was associated with a 26-31% reduction in odds of mortality compared to surgical aortic valve replacement in women, there was no difference in mortality between TAVR and surgical aortic valve replacement in men (51). Besides, the female gender has been reported to correlate with a better short- and long-term post-TAVR survival (52). Nevertheless, because most of our included studies only provided the gender proportions of their participants without separating the two genders for outcome comparison, we could not conduct a gender-based subgroup analysis on our included outcomes. Finally, variations in operator experience (48) and clinical protocols in our included studies, which may introduce bias to our results, were not analyzed in the current investigation.

In conclusion, compared with general anesthesia, our results demonstrated an association of conscious sedation/monitored anesthesia with a reduced hospital length of stay and possibly lower risks of 30-day and one-year mortality, acute kidney injury, major bleeding as well as shorter procedural time and ICU stay. However, the current meta-analysis of observational studies cannot establish the causality of our findings, which requires further randomized controlled trials for elucidation.

## Data availability statement

The original contributions presented in this study are included in this article/Supplementary material, further inquiries can be directed to the corresponding author.

## Author contributions

K-CH and J-YC: conceptualization, methodology, and software. C-HH and C-CC: data curation. K-CH, I-CT, and Y-LP: writing – original draft preparation. Y-TL and I-WC: visualization and investigation. C-KS: supervision. K-CH and I-WC: software and validation. K-CH and C-KS: writing – reviewing and editing. I-WC and C-KS contributed equally as corresponding authors to this work. All authors contributed to the article and approved the submitted version.
