# Supplementary material for: Conscious sedation/monitored anesthesia care versus general anesthesia in patients undergoing transcatheter aortic valve replacement: A meta-analysis
Source: Front Cardiovasc Med. 2023 Jan 10;9:1099959. doi: 10.3389/fcvm.2022.1099959 (PMC9872395; doi:10.3389/fcvm.2022.1099959)
Supplement: Supplementary file 1 [file Table_1.DOCX]

Supplemental materials

**Supplemental Table 1.** Search strategies for databases

**Supplemental Table 2.** Details for assessment of paravalvular leak in individual study

**Supplemental Table 3.** Quality of included studies assessed with Newcastle Ottawa scale (n = 22)

**Supplemental Table 4**. Risk of bias domains for randomized controlled trials

**Supplemental table 5.** Summary of findings for the main comparison

**Supplemental figure 1.** Forest plot showing difference in Society of Thoracic Surgeons (STS) predicted risk for mortality score in patients receiving conscious sedation/monitored anesthesia care (CS/MAC) or general anesthesia (GA).

**Supplemental figure 2.** Forest plot showing conversion rate in patients receiving conscious sedation/monitored anesthesia care.

**Supplemental figure 3.** Funnel plot showing a low risk of publication bias regarding the outcome on risk of 30-day mortality.

**Supplemental figure 4.** Forest plot comparing the risk of one-year mortality in patients receiving conscious sedation/monitored anesthesia care (CS/MAC) or general anesthesia (GA). CI: confidence interval; M-H: Mantel-Haenszel.

**Supplemental figure 5.** Forest plot comparing the risk of myocardial infarction in patients receiving sedation/monitored anesthesia care (CS/MAC) or general anesthesia (GA). CI: confidence interval; M-H: Mantel-Haenszel.

**Supplemental figure 6.** Forest plot comparing the risk of pacemaker implantation in patients receiving sedation/monitored anesthesia care (CS/MAC) or general anesthesia (GA). CI: confidence interval; M-H: Mantel-Haenszel.

**Supplemental figure 7.** Funnel plot showing a low risk of publication bias regarding the outcome on the pacemaker implantation risk.

**Supplemental figure 8.** Forest plot comparing the procedural time in patients receiving sedation/monitored anesthesia care (CS/MAC) or general anesthesia (GA). CI: confidence interval.

**Supplemental figure 9.** Funnel plot showing a low risk of publication bias regarding the outcome on the procedural time.

**Supplemental figure 10.** Forest plot comparing the risk of vascular complications in patients receiving sedation/monitored anesthesia care (CS/MAC) or general anesthesia (GA). CI: confidence interval.

**Supplemental figure 11.** Funnel plot showing a low risk of publication bias regarding the outcome on the risk of vascular complications.

**Supplemental figure 12.** Forest plot comparing the risk of major bleeding in patients receiving sedation/monitored anesthesia care (CS/MAC) or general anesthesia (GA). CI: confidence interval.

**Supplemental figure 13.** Funnel plot showing a low risk of publication bias regarding the outcome on the risk of major bleeding.

**Supplemental figure 14.** Forest plot comparing the procedural success rate in patients receiving sedation/monitored anesthesia care (CS/MAC) or general anesthesia (GA). CI: confidence interval.

**Supplemental figure 15.** Funnel plot showing a low risk of publication bias regarding the outcome on the risk of stroke.

**Supplemental figure 16.** Forest plot comparing the risk of acute kidney injury in patients receiving sedation/monitored anesthesia care (CS/MAC) or general anesthesia (GA). CI: confidence interval.

**Supplemental figure 17.** Funnel plot showing a low risk of publication bias regarding the outcome on the risk of acute kidney injury.

**Supplemental figure 18.** Forest plot comparing the intensive care unit length of stay in patients receiving sedation/monitored anesthesia care (CS/MAC) or general anesthesia (GA). CI: confidence interval.

**Supplemental figure 19.** Funnel plot showing a low risk of publication bias regarding the outcome on the intensive care unit length of stay.

**Supplemental figure 20.** Funnel plot showing a low risk of publication bias regarding the outcome on the hospital length of stay.

**Supplemental Table 1.** Search strategies for databases

| Database |  | Key words or MeSH terms |
| --- | --- | --- |
| **MEDLINE (Ovid)** | #1 | ("transcatheter aortic valve replacement" or "TAVR" or "Aortic valve stenosis" or "Transcatheter Aortic Valve Implantation" or "TAVI").mp |
|  | #2 | exp "Transcatheter Aortic Valve Replacement"/ or exp "Aortic Valve Stenosis"/ |
|  | #3 | ("General anesthesia" or "Tracheal intubation*" or "Endotracheal Intubation").mp |
|  | #4 | exp "Anesthesia, General"/ or exp "Intubation, Intratracheal"/ |
|  | #5 | ("Sedation" or "Monitored anesthesia care").mp |
|  | #6 | exp "Deep Sedation"/ or exp "Conscious Sedation"/ |
|  | #7 | (1 or 2) and (3 or 4) and (5 or 6) |
| **Embase (Ovid)** | #1 | ("transcatheter aortic valve replacement" or "TAVR" or "Aortic valve stenosis" or "Transcatheter Aortic Valve Implantation" or "TAVI").mp |
|  | #2 | exp "Transcatheter Aortic Valve Replacement"/ or exp "Aortic Valve Stenosis"/ |
|  | #3 | ("General anesthesia" or "Tracheal intubation*" or "Endotracheal Intubation").mp |
|  | #4 | exp "Anesthesia, General"/ or exp "Intubation, Intratracheal"/ |
|  | #5 | ("Sedation" or "Monitored anesthesia care").mp |
|  | #6 | exp "Deep Sedation"/ or exp "Conscious Sedation"/ |
|  | ***#7*** | (1 or 2) and (3 or 4) and (5 or 6) |
| **Cochrane CENTRAL** | #1 | ("transcatheter aortic valve replacement" or "TAVR" or "Aortic valve stenosis" or "Transcatheter Aortic Valve Implantation" or "TAVI"):ti,ab,kw |
|  | #2 | [mh "Transcatheter Aortic Valve Replacement"] or [mh "Aortic Valve Stenosis"] |
|  | #3 | ("General anesthesia" or "Tracheal intubation*" or "Endotracheal Intubation"):ti,ab,kw |
|  | #4 | [mh "Anesthesia, General"] or [mh "Intubation, Intratracheal"] |
|  | #5 | ("Sedation" or "Monitored anesthesia care"):ti,ab,kw |
|  | #6 | [mh "Deep Sedation"] OR [mh "Conscious Sedation"] |
|  | #7 | (#1 or #2) and (#3 or #4) and (#5 or #6) |

**Supplemental Table 2.** Details for assessment of paravalvular leak in patients receiving sedation or monitored anesthesia care

| Study name | Techniques for assessment of paravalvular leak in patients receiving sedation |
| --- | --- |
| Abbett 2021 | NR |
| Ahmad 2019 | NR |
| Brecker 2016 | NR |
| Burns 2019 | Transesophageal echocardiography, performed by an interventional echocardiography cardiologist, was replaced with transthoracic imaging performed principally by a sonographer |
| Butala 2020 | NR |
| D'Errigo 2016 | NR |
| Eskandari 2018 | Aortic regurgitation (AR) at the end of procedure was assessed by transoesophageal echocardiography (TOE), transthoracic echocardiography (TTE) or aortic root angiography and graded as mild, moderate and severe. |
| Feistritzer 2021 | NR |
| Goren 2015 | NR |
| Herrmann 2021 | NR |
| Hyman 2017 | NR |
| Harjai 2020 | Aortic regurgitation (AR) at the end of procedure was assessed by transthoracic echocardiography (TTE) |
| Kiramijyan 2016 | In the cases with sedation, the transesophageal echocardiography probe was inserted after sedation through the bite-block. Intra-procedural transthoracic echocardiography was also completed for a minority of the cases. |
| Kislitsina 2019 | Transesophageal echocardiography was not used in sedated patients and valve deployment was performed under fluoroscopic guidance. |
| Lau 2020 | Transthoracic echocardiography (TTE) was performed in patients receiving sedation, while transesophageal echocardiography (TEE) was performed in all patients receiving general anesthesia. |
| Lee 2021 | After valve placement, the function and optimal position of the valve were confirmed by transesophageal echocardiography in patients in the GA group, by transthoracic echocardiography (TTE) in patients in the MAC group, and by fluoroscopy in both groups. |
| Lum 2021 | Transthoracic echocardiography (TTE) was performed in sedation group. |
| Mosleh 2019 | NR |
| Musuku 2021 | If a patient in the sedation group required transesophageal echocardiography and was hemodynamically stable, it was performed using sedation. |
| Neumann 2020 | NR |
| Renner 2019 | Transthoracic echocardiography was performed prior to the start of intervention and at the end of the procedure. |
| Sammour 2021 | NR |
| Thiele 2020 | Assessment of periprocedural TAVR results was performed according to local practice by angiographic, hemodynamic, and in some cases, echocardiographic, evaluation of valves. |
| Zaouter 2018 | Valves were deployed under fluoroscopic guidance in the sedation group and by fluoroscopy and transesophageal echocardiography in the GA group. |

NR: not reported

Supplemental Table 3. Quality of included studies assessed with Newcastle Ottawa scale^§^ (n = 22)

| Study |  | Number of stars awarded in each domain | | |  |  |
| --- | --- | --- | --- | --- | --- | --- |
|  |  | Selection  (Maximum: 4★) | Comparability  (Maximum: 2★) | Outcome  (Maximum: 3★) |  | Total score  (Out of 9) |
| Abbett 2021 |  | ★★★ | ★★ | ★★★ |  | 8 |
| Ahmad 2019 |  | ★★★ | ★ | ★★★ |  | 7 |
| Brecker 2016 |  | ★★★ | ★★ | ★★★ |  | 8 |
| Burns 2019 |  | ★★★ | ★★ | ★★★ |  | 8 |
| Butala 2020 |  | ★★★ | ★★ | ★★★ |  | 8 |
| D'Errigo 2016 |  | ★★★ | ★★ | ★★★ |  | 8 |
| Eskandari 2018 |  | ★★★ | ★★ | ★★★ |  | 8 |
| Goren 2015 |  | ★★★ | ★ | ★★★ |  | 7 |
| Herrmann 2021‡‡ |  | ★★★ | ★ | ★★★ |  | 7 |
| Herrmann 2021‡‡ |  | ★★★ | ★ | ★★★ |  | 7 |
| Hyman 2017 |  | ★★★ | ★ | ★★★ |  | 7 |
| Harjai 2020 |  | ★★★ | ★ | ★★★ |  | 7 |
| Kiramijyan 2016 |  | ★★★ | ★ | ★★★ |  | 7 |
| Kislitsina 2019 |  | ★★★ | ★★ | ★★★ |  | 8 |
| Lau 2020 |  | ★★★ | ★★ | ★★★ |  | 8 |
| Lee 2021 |  | ★★★ | ★ | ★★★ |  | 7 |
| Lum 2021 |  | ★★★ | ★★ | ★★★ |  | 8 |
| Mosleh 2019 |  | ★★★ | ★★ | ★★★ |  | 8 |
| Musuku 2021 |  | ★★★ | ★★ | ★★★ |  | 8 |
| Neumann 2020 |  | ★★★ | ★ | ★★★ |  | 7 |
| Renner 2019 |  | ★★★ | ★★ | ★★★ |  | 8 |
| Sammour 2021 |  | ★★★ | ★ | ★★★ |  | 7 |
| Zaouter 2018 |  | ★★★ | ★ | ★★★ |  | 7 |

^§^A maximum of four, two, and three stars assigned to the selection, comparability, and outcome domains, respectively. The higher the number of stars, the better the quality of the study. ‡‡Two dataset available

**Supplemental Table 4**. Risk of bias domains for randomized controlled trials

| Study | D1 | D2 | D3 | D4 | D5 | Overall |
| --- | --- | --- | --- | --- | --- | --- |
| Feistritzer 2021 | Low | Low | Low | Low | Low | Low |
| Thiele 2020 | Low | Low | Low | Low | Low | Low |

Domains:

D1: Bias arising from the randomization process

D2: Bias due to deviations from intended intervention

D3: Bias due to missing outcome data.

D4: Bias in measurement of the outcome.

D5: Bias in selection of the reported result.

**Supplemental table 5.** Summary of findings for the main comparison

|  | | | | | | |
| --- | --- | --- | --- | --- | --- | --- |
| Outcomes | Effect (Risk or mean) | | Relative effect (95% CI) | № of participants  (studies) | Certainty of the evidence (GRADE) | Comments |
|  | Intervention group | Control group |  |  |  |  |
| Risk of 30-day mortality | 1324/71717 | 1952/68014 | OR 0.66 (0.62 to 0.71) | 139731 (21 studies) | ⨁⨁◯◯ Low | a |
| Risk of one-year mortality | 273/2448 | 290/2379 | OR 0.72  (0.59 to 0.88) | 4827 (9 studies) | ⨁◯◯◯ Very Low | a, b |
| Risk of vasopressor/inotropic support | 18044/68302 | 22926/65136 | OR 0.25 (0.17 to 0.38) | 133438 (9 studies) | ⨁◯◯◯ Very Low | a, c |
| Risk of stroke | 113/5308 | 109/4494 | OR 0.81  (0.6 to 1.09) | 9802  (20 studies) | ⨁◯◯◯ Very Low | a, b |
| Risk of myocardial infarction | 11/2614 | 9/2216 | OR 1.07  (0.43 to 2.65) | 4830  (8 studies) | ⨁◯◯◯ Very Low | a, b |
| Risk of pacemaker implantation | 779/6114 | 1566/12402 | OR 1.05  (0.9 to 1.22) | 18516  (16 studies) | ⨁⨁◯◯ Low | a |
| Risk of vascular complications | 253/3991 | 159/2939 | OR 1.1  (0.89 to 1.37) | 6930  (13 studies) | ⨁◯◯◯ Very Low | a, b |
| Risk of major bleeding | 230/3750 | 348/3138 | OR 0.61  (0.41 to 0.9) | 6888  (13 studies) | ⨁◯◯◯ Very Low | a, b, c |
| Procedural success rate | 65942/67642 | 63098/64749 | OR 1.02  (0.71 to 1.47) | 132391  (7 studies) | ⨁◯◯◯ Very Low | a, b, c |
| Risk of acute kidney injury | 114/4035 | 146/3120 | OR 0.71  (0.54 to 0.92) | 7155  (15 studies) | ⨁◯◯◯ Very Low | a, b |
| Hospital length of stay | - | - | MD -0.84 (-0.98 to -0.7) | 19019  (17 studies) | ⨁◯◯◯ Very Low | a, b, c |
| Procedural time | - | - | MD -12.27  (-19.24 to -5.31) | 17694  (15 studies) | ⨁◯◯◯ Very Low | a, b, c |
| Intensive care unit length of stay | - | - | MD -7.53  (-14.82 to -0.25) | 7589  (14 studies) | ⨁◯◯◯ Very Low | a, b, c |

Comments:

^a^Evidence that includes observational data starts at low quality.

^b^wide 95% confidence interval

^c^The I square is more than 50%.

GRADE Working Group grades of evidence:
-High certainty: We are very confident that the true effect lies close to that of the estimate of the effect
-Moderate certainty: We are moderately confident in the effect estimate: The true effect is likely to be close to the estimate of the effect, but there is a possibility that it is substantially different.
-Low certainty: Our confidence in the effect estimate is limited: The true effect may be substantially different from the estimate of the effect.
-Very low certainty: We have very little confidence in the effect estimate: The true effect is likely to be substantially different from the estimate of effect.

**Supplemental figure 1.** Forest plot showing difference in Society of Thoracic Surgeons (STS) predicted risk for mortality score in patients receiving conscious sedation/monitored anesthesia care or general anesthesia.


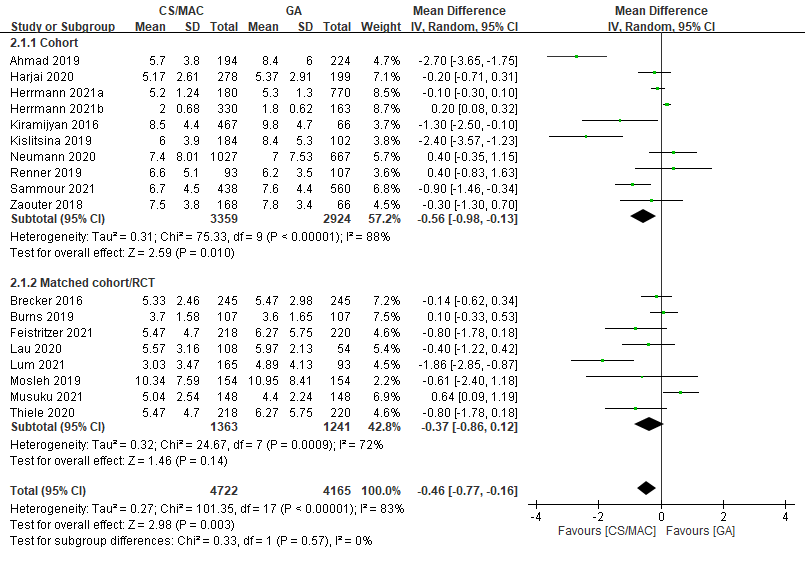


**Supplemental figure 2.** Forest plot showing conversion rate in patients receiving conscious sedation/monitored anesthesia care (CS/MAC).


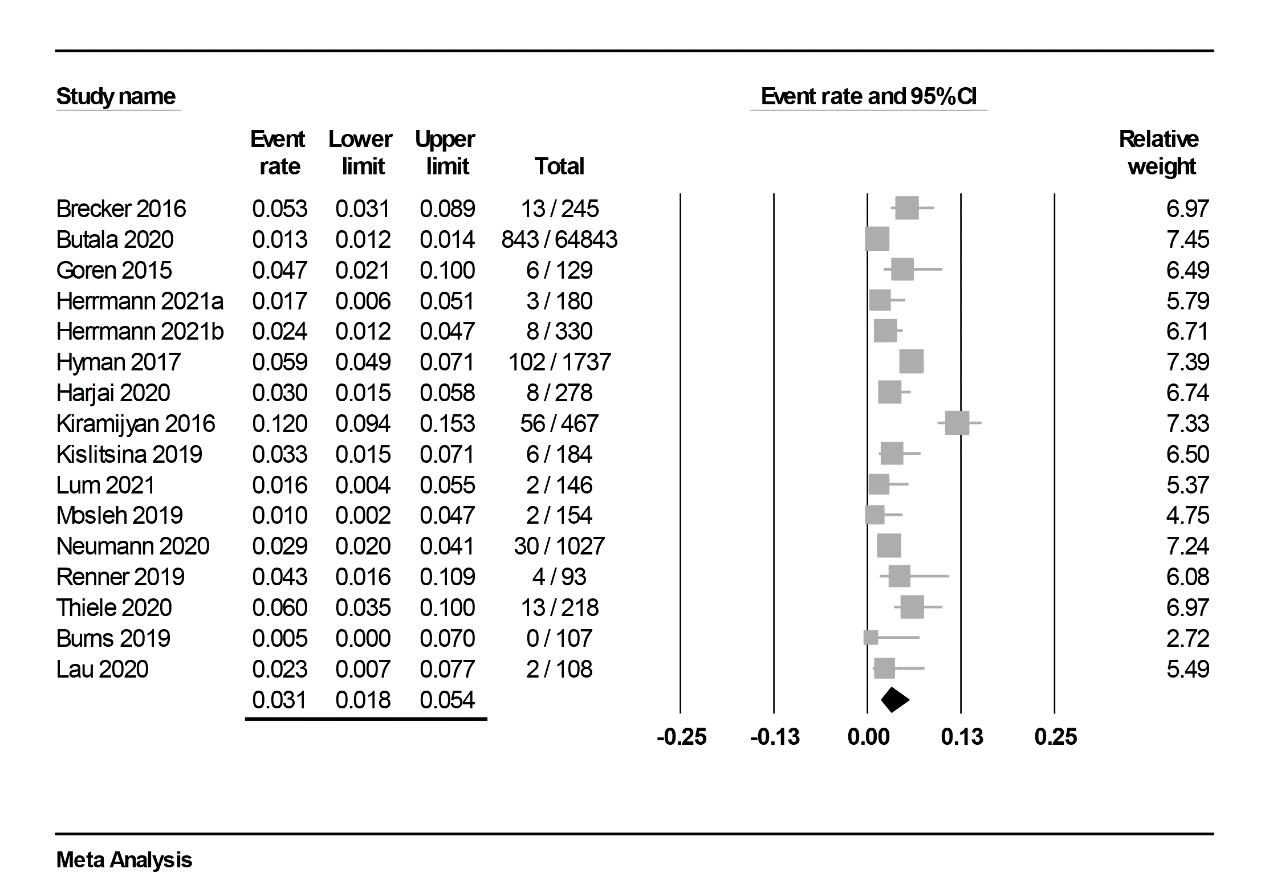


**Supplemental figure 3.** Funnel plot showing a low risk of publication bias regarding the outcome on risk of 30-day mortality.

**
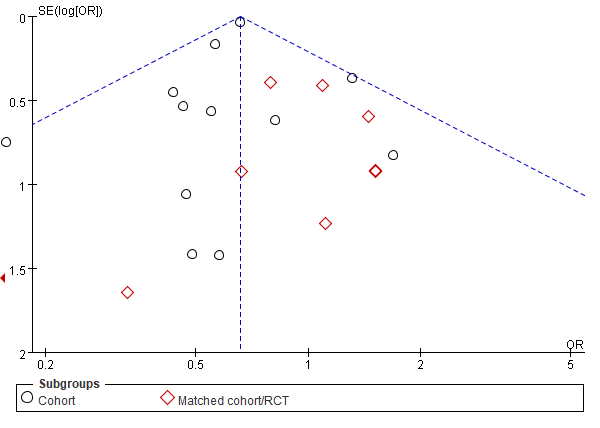
**

**Supplemental figure 4.** Forest plot comparing the risk of one-year mortality in patients receiving conscious sedation/monitored anesthesia care (CS/MAC) or general anesthesia (GA). CI: confidence interval; M-H: Mantel-Haenszel.


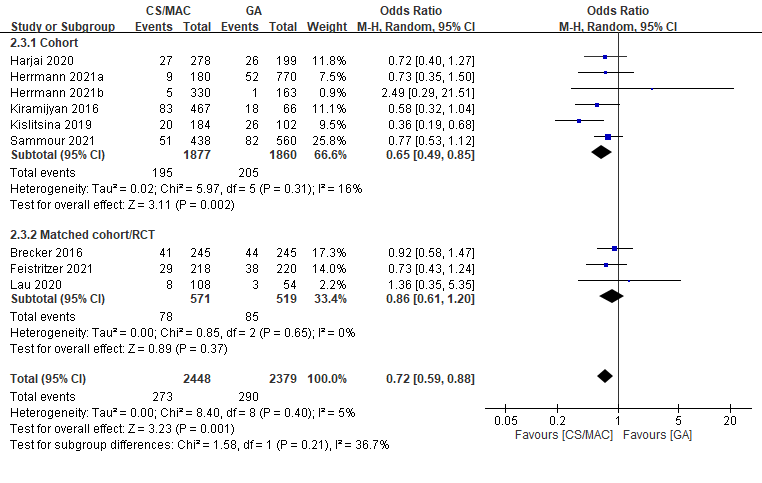


**Supplemental figure 5.** Forest plot comparing the risk of myocardial infarction in patients receiving sedation/monitored anesthesia care (CS/MAC) or general anesthesia (GA). CI: confidence interval; M-H: Mantel-Haenszel.


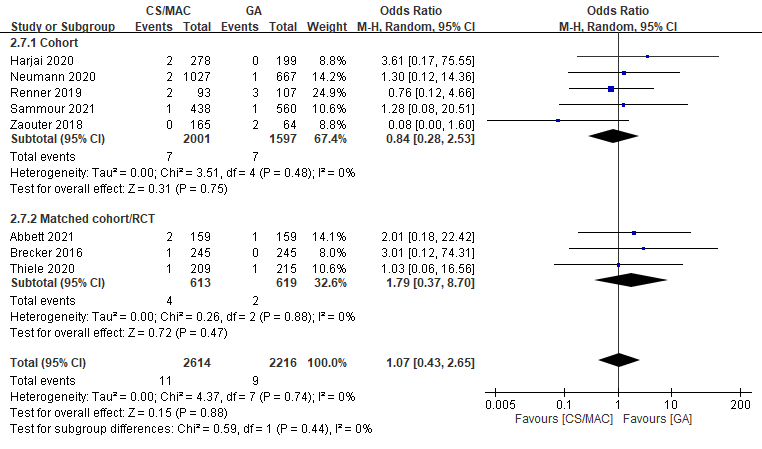


**Supplemental figure 6.** Forest plot comparing the risk of pacemaker implantation in patients receiving sedation/monitored anesthesia care (CS/MAC) or general anesthesia (GA). CI: confidence interval; M-H: Mantel-Haenszel.


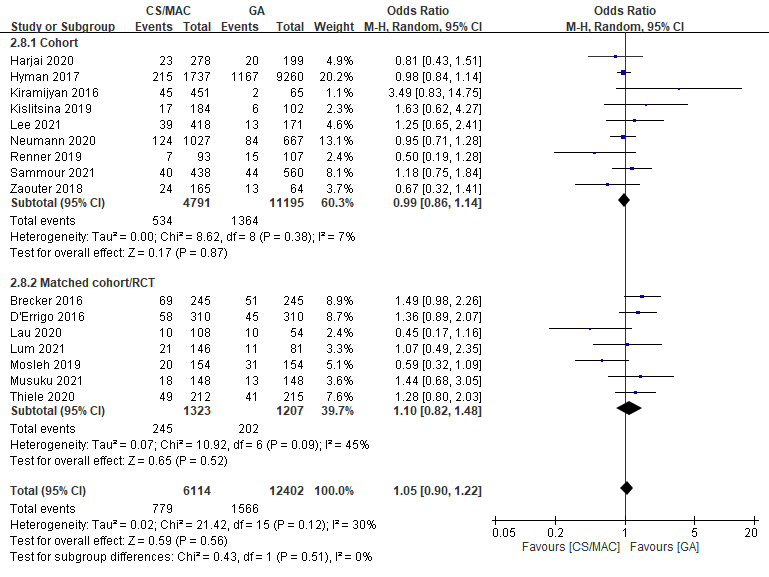


**Supplemental figure 7.** Funnel plot showing a low risk of publication bias regarding the outcome on the risk of pacemaker implantation.


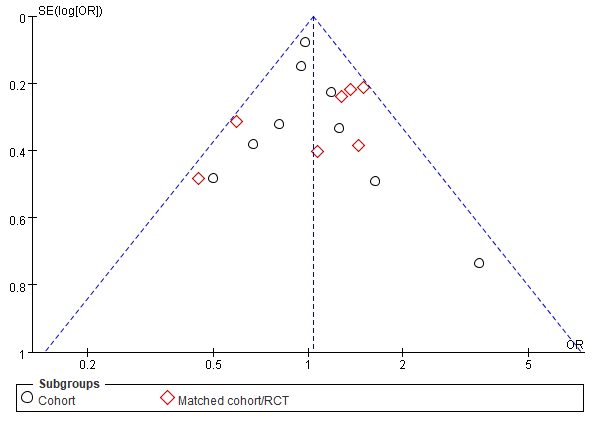


**Supplemental figure 8.** Forest plot comparing the procedural time in patients receiving sedation/monitored anesthesia care (CS/MAC) or general anesthesia (GA). CI: confidence interval.


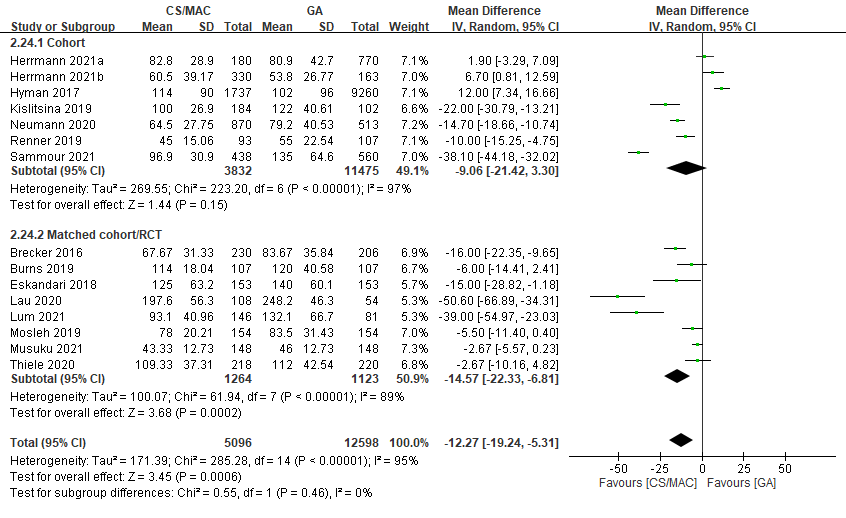


**Supplemental figure 9.** Funnel plot showing a low risk of publication bias regarding the outcome on the procedural time.


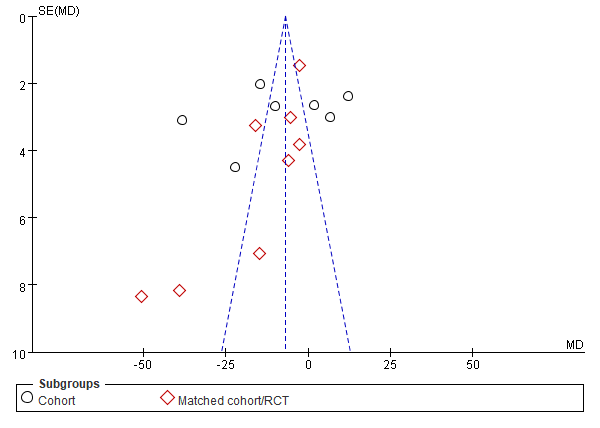


**Supplemental figure 10.** Forest plot comparing the risk of vascular complications in patients receiving sedation/monitored anesthesia care (CS/MAC) or general anesthesia (GA). CI: confidence interval.


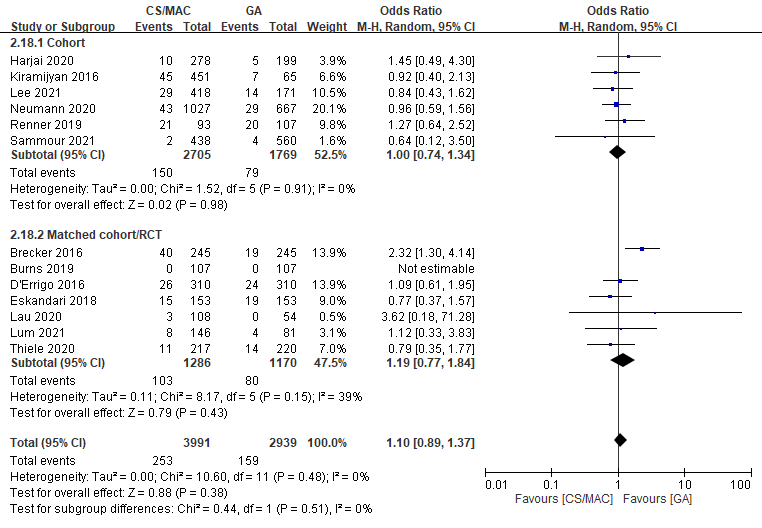


**Supplemental figure 11.** Funnel plot showing a low risk of publication bias regarding the outcome on the risk of vascular complications.


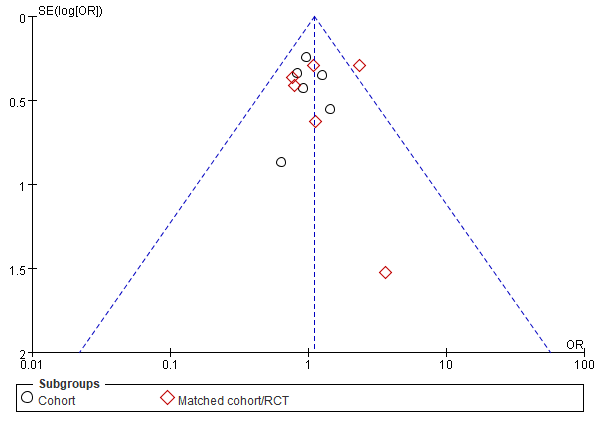


**Supplemental figure 12.** Forest plot comparing the risk of major bleeding in patients receiving sedation/monitored anesthesia care (CS/MAC) or general anesthesia (GA). CI: confidence interval.

**
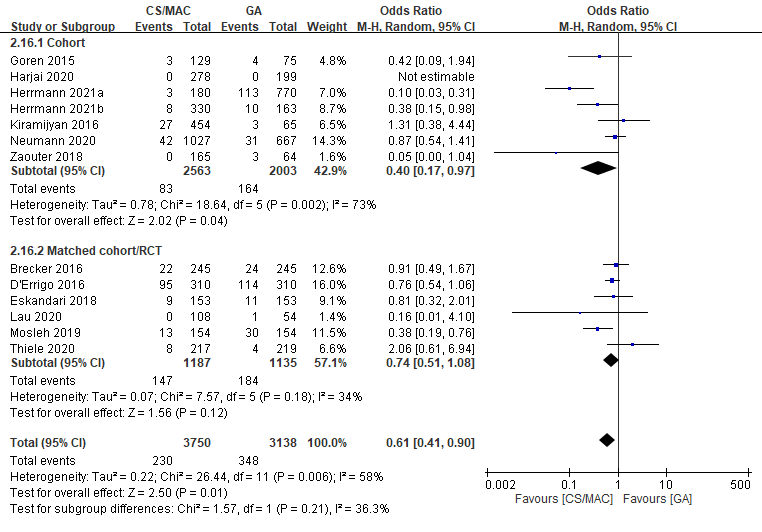
**

**Supplemental figure 13.** Funnel plot showing a low risk of publication bias regarding the outcome on the risk of major bleeding.


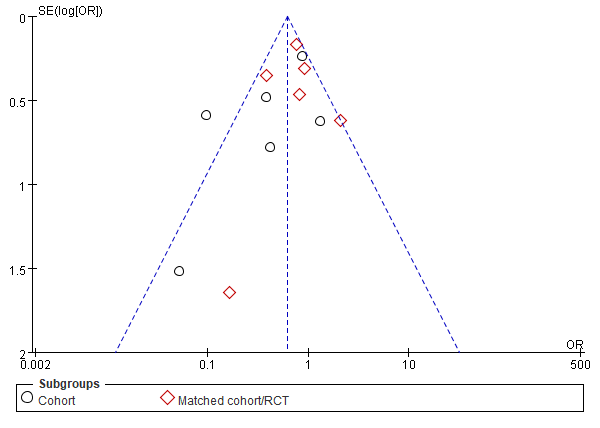


**Supplemental figure 14.** Forest plot comparing the procedural success rate in patients receiving sedation/monitored anesthesia care (CS/MAC) or general anesthesia (GA). CI: confidence interval.

**
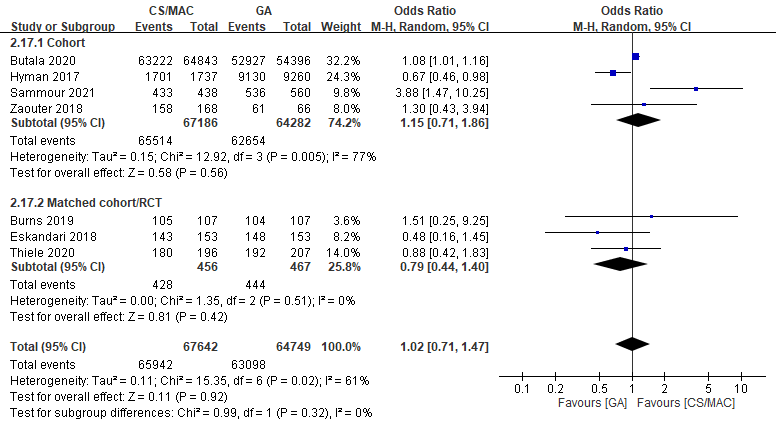
**

**Supplemental figure 15.** Funnel plot showing a low risk of publication bias regarding the outcome on the risk of stroke.


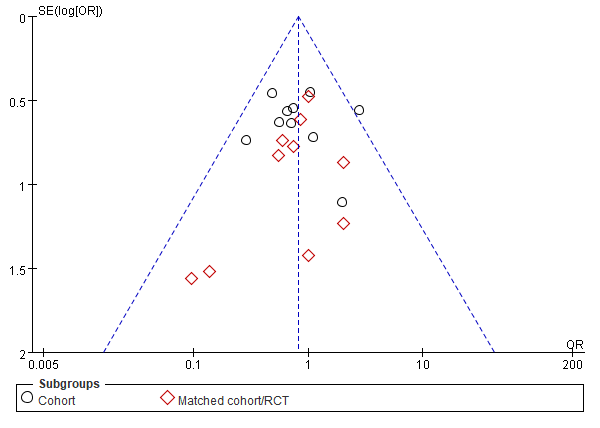


**Supplemental figure 16.** Forest plot comparing the risk of acute kidney injury in patients receiving sedation/monitored anesthesia care (CS/MAC) or general anesthesia (GA). CI: confidence interval.

**
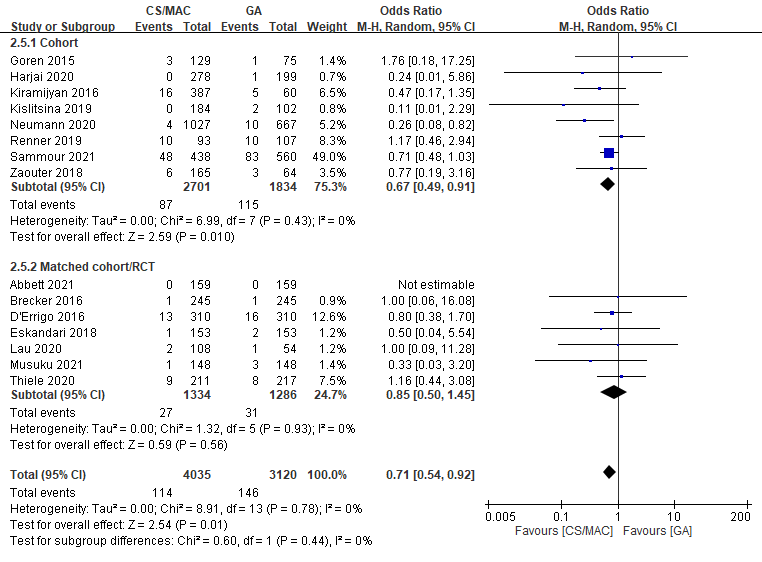
**

**Supplemental figure 17.** Funnel plot showing a low risk of publication bias regarding the outcome on the risk of acute kidney injury.

**
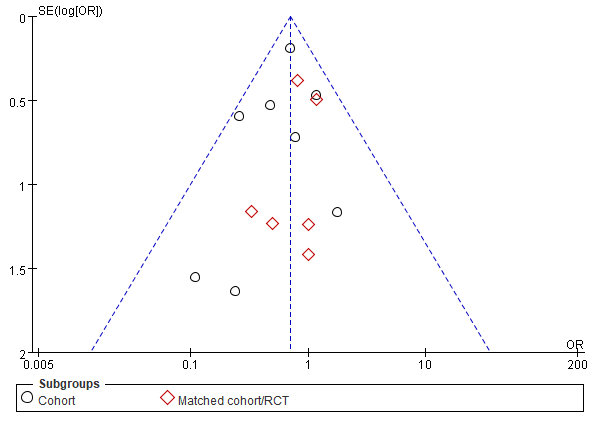
**

**Supplemental figure 18.** Forest plot comparing the intensive care unit length of stay in patients receiving sedation/monitored anesthesia care (CS/MAC) or general anesthesia (GA). CI: confidence interval.

**
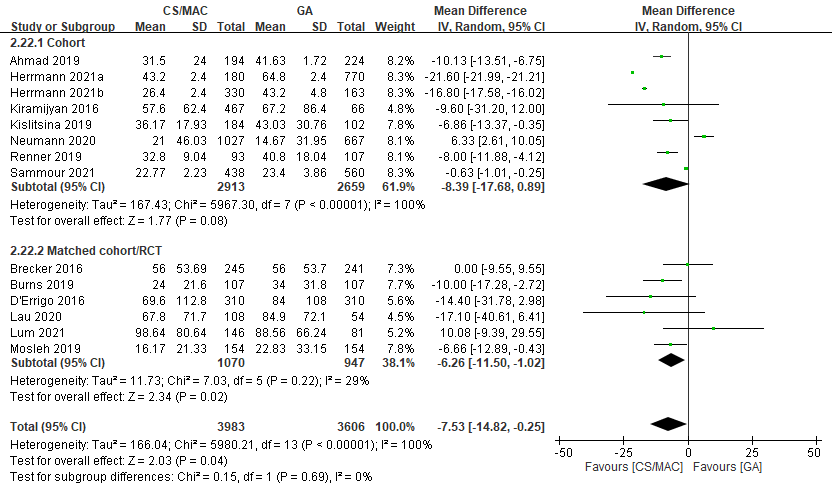
**

**Supplemental figure 19.** Funnel plot showing a low risk of publication bias regarding the outcome on the intensive care unit length of stay.

**
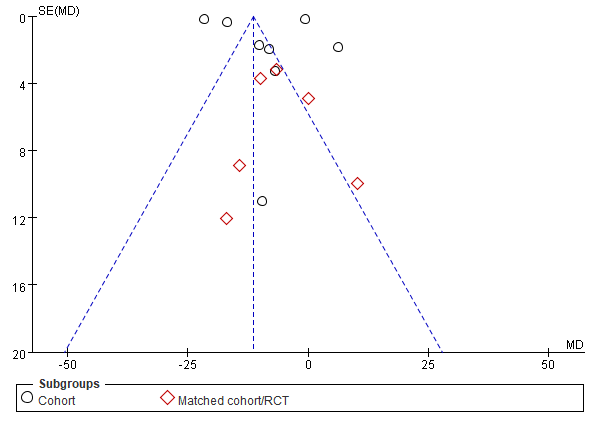
**

**Supplemental figure 20.** Funnel plot showing a low risk of publication bias regarding the outcome on the hospital length of stay.

**
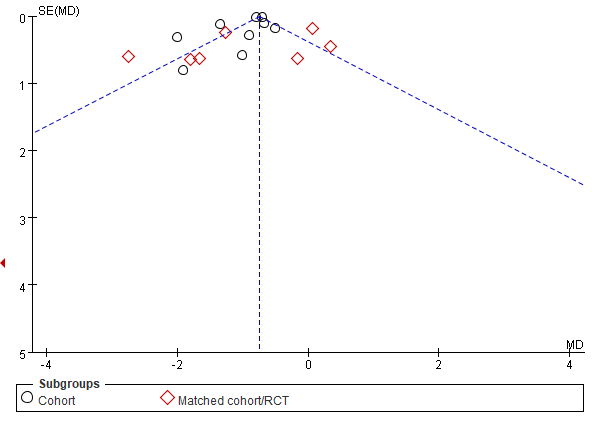
**
